# Antiarthritic activities of berberine in a rat model of gouty arthritis

**DOI:** 10.1038/s41598-025-16622-0

**Published:** 2025-09-01

**Authors:** Ghadha Ibrahim Fouad, Hanan F. Aly, Mohamed B. Shalaby, Mohamed I. Mabrouk, Wagdy K. B. Khalil, Maha Z. Rizk

**Affiliations:** 1 Department of Therapeutic Chemistry, National Research Centre, 33 El-Bohouth St., Dokki, Cairo, 12622 Egypt; 2 Department of Toxicology Research, Research Institute of Medical Entomology (RIME), General Organization of Teaching Hospitals and Institutes (GOTHI), Ministry of Health and Population (MoHP), Dokki, Cairo, 12311 Egypt; 3 Faculty of Applied Medical Sciences, Applied Science Private University, Amman, Jordan; 4 Department of Cell Biology, National Research Centre, 33 El-Bohouth St., Dokki, Cairo, 12622 Egypt

**Keywords:** Gouty arthritis, Monosodium urate, Berberine, DNA fragmentation, Inflammation, Biochemistry, Cell biology, Molecular biology, Biomarkers

## Abstract

Gouty arthritis (GA) is an inflammatory arthritic disorder that is characterized by intense, acute inflammatory responses, such as synovitis and arthritis that occur due to articular deposition of monosodium urate (MSU) crystals. This study has compared the therapeutic potentials of either Berberine (BERB) or Paracetamol (Para) on MSU-induced inflammation in rat model of Gouty arthritis (GA). GA was induced by “intra-articular” injection of MSU suspension (20 mg/ml) inside the knee joint of the rat’s right limb. Circumference was measured at 48 h after MSU injection and 14 days post-treatment with BERB or Para. Gait assessment was conducted. Histopathological alterations of knee and paw (ankle) joint tissue were investigated. Serum levels of Malondialdehyde (MDA), monocyte chemotactic protein 1 (MCP-1), Vascular endothelial growth factor (VEGF), and prostaglandin E2 (PGE2) were estimated. Molecular analysis of Elastase, Cyclooxygenase-2 (COX-2), Matrix metalloproteinase-9 (MMP-9), and Myeloperoxidase (MPO) was evaluated. In addition, DNA fragmentation assay was performed. Our results revealed that deposition of MSU crystals in the articular joints provoked an inflammatory response, oxidative stress, and DNA fragmentation (apoptosis). However, the oral treatment of MSU-induced rats with either BERB (50 mg/kg/day) or Para (50 mg/kg/day) for 14 days mitigated MSU-stimulated inflammation and arthritis as represented by the behavioral, histopathological, biochemical, and molecular levels. Treatment of MSU-induced arthritic rats with BERB or Para attenuated oedema and alleviated histological signs of acute inflammation. In addition, treatment decreased chemokine levels and reduced MDA levels. These results indicated that BERB and Para exerted strong anti-inflammatory, anti-oxidative, and anti-apoptotic activities against GA.

**Supplementary Information:**

The online version contains supplementary material available at 10.1038/s41598-025-16622-0.

## Introduction

Gouty arthritis (GA) is a progressive and age-related “osteoarticular disease” that is characterized by joint pain and dysfunction; due to articular cartilage degeneration and peri-articular hyperostosis through affecting the articular cartilage, bone and synovial tissue^1,2^.

GA is an inflammatory arthritis caused by the deposition of needle-like monosodium urate (MSU) crystals in cartilage, bone, and tissues around the joint, due to dysfunction in purine metabolism and uric acid excretion^3,4^. Accumulated MSU crystals in the articular joints stimulate the production of inflammatory mediators “cytokines, chemokines, proteases, and oxidants”; thereby amplifying the inflammatory response manifested as synovitis, arthritis, cartilage loss, and bone resorption^5^. This painful type of arthritis “rheumatoid joint disease” results in joint deformation and is often associated with several clinical complications, such as diabetes, high blood pressure, and cardiovascular disorders^6^.

The hallmarks of GA attacks include edema (vasodilation), erythema, and severe joint pain; therefore the pharmacological treatment should provide prompt and short-term symptomatic relief to ensure alleviation of articular pain and inflammation, and amelioration of edema^7,8^. Currently, non-steroidal anti-inflammatory drugs (NSAIDs), glucocorticoids, and colchicine are used to shorten the gouty attack; however; the current used drugs are not efficient due to several factors such as gastrointestinal irritation, drug resistance, increased risk for serious cardiovascular events, and other side effects^9,10^. Moreover, NSAIDs are not effective enough to exhibit long-term outcomes in osteoarthritis (OA) and rheumatoid arthritis (RA). Thereby, it is recommended to use low doses of NSAIDs for a short-term period^11^.

Paracetamol “acetaminophen, N-acetyl-p-aminophenol”, exhibits a superior safety profile to NSAIDs, is a widely used analgesic, to mitigate mild to moderate chronic pain associated with OA^12^; the clinical efficacy of Paracetamol (Para) is equivalent to NSAIDs and only slightly inferior for all musculoskeletal pains. Therefore, Para is recommended for first-line analgesia for amelioration of RA or OA^9^. In addition, Para is safe to use and is an effective alternative to aspirin^13^.

Para is safe if taken at therapeutic doses, however is commonly taken in overdose, where it can provoke hepatotoxicity^14^. Para overdose-induced hepatotoxicity is the most common cause of Drug induced liver injury (DILI); Para-induced hepatotoxicity is generally defined as either AST or ALT > 1000 IU/L^14,15^. It is also a direct mitochondrial toxin and at very high concentrations can cause central nervous system (CNS) depression^14^. Given the increased prevalence of combination medications in the form of pain relievers and antihistamines, Para can be difficult to be identified and remains a main factor of acute hepatotoxicity^15^.

In this concern, it is essential to find nature-based effective therapeutic agents for alleviating GA, with demonstrated efficacy in reducing GA duration or improving recovery while minimizing liver and kidney damage. The extracts of natural herbs demonstrated promising therapeutic potentials, less side effects, and availability^16^. A plethora of phytochemicals exhibited anti-oxidative and anti-inflammatory activities against several disorders^17–20^.

Berberine (BERB) is a natural iso-quinoline alkaloid extracted from *Coptidis* rhizome and *Cortex phellodendri*, which is an important traditional Chinese medicinal herb^20^. BERB has been previously reported to exhibit several pharmacological functions and thus could be used to treat several disorders^20–24^. The anti-inflammatory activity of BERB could be explained by its potential to regulate “miR-181c-5p/HMGB1” axis or inhibit the “HMGB1/TLR4/NF-κB” pathway^25^.

Furthermore, BERB exerts its anti-inflammatory activities through different pathways, such as: (1) Suppressing the synthesis and production of inflammatory cytokines (*e.g.* tumor necrosis factor-α (TNF-α), Interleukin-1 beta (IL-1β), Interleukin-6 (IL-6)) through inhibiting the activation of the NF-κB signaling pathway^26–28^. (2) Deactivating the Toll-like receptor 4/nuclear factor-kappa B (TLR4/NF-κB) signaling and inhibiting the activation of the pyrin domain-containing-3 (NLRP3) inflammasome pathway possibly by regulating the axis of mTOR/mtROS to inhibit pyroptosis, and protecting the mitochondrial integrity^29,30^. (3) Exerting anti-oxidative potential and protecting cellular functions and integrity^31^. (4) Exerting immunomodulating functions^32^ and suppressing leukocyte adhesion and chemotaxis^33^.

These activities support the potential of BERB as a multifactorial therapeutic molecule for bone-related disorders. Regarding the role of BERB in the treatment of RA, a recent study by Wang et al.^34^, using network pharmacology, revealed that “the ten core targets with high DEGREE values” includes IL-10, IL-4, IL-1β, TNF-α, and IL-6.

The aim of the current study is to investigate the therapeutic and alleviative potentials of either BERB or Para on the levels of inflammatory mediators in the peripheral blood of MSU-induced GA rats; and hence to provide a theoretical basis for clinical treatment of this osteoarticular disease.

## Materials and methods

### Chemicals and reagents

Uric acid sodium salt (U2875-5G) and Berberine chloride hydrate (BERB) were purchased from Sigma Aldrich (St. Louis, MO, USA) and dissolved in distilled water. Paracetamol (Para) was purchased from a local pharmacy in Egypt. Malondialdehyde (MDA) was measured colorimetrically using Biodiagnostic assay kit, Egypt. Other kits were bought from Endogen, Rockford, USA. QuantiTect real time-PCR Kits (Cat no. ID. 204443) and RNeasy Kits (Cat no. ID. 74104) were bought from QIAGEN, (Amtsgericht Düsseldorf, Germany). Primers of Elastase, COX-2, MMP-9, and MPO were purchased from Qiagen (Amtsgericht Düsseldorf, Germany).

### Synthesis of MSU crystals

MSU crystals were prepared by re-crystallization from uric acid according the previously reported method^35^.

### Animals

Twenty four male white albino rats (150 ± 20 g; 6–8 weeks) were purchased from the Animal house of the National Research Centre (NRC, Egypt). The rats were allocated randomly into four groups and housed in stainless steel cages (n = 6 per group). The rats were allowed one week for adaptation before the start of the experimental period. Rats were administrated freely accessible standard chow and water. All care and procedures used in the experiments were in accordance with the ARRIVE guidelines and approved by the “Medical Research Ethics Committee (MREC) at the National Research Centre (NRC)” (no. 04440425). Additionally, all methods were performed in accordance with the relevant guidelines and regulations.

### MSU-induced joint inflammation and gouty arthritis (GA) in rats

The experimental GA rat model was induced using MSU crystals. To stimulate joint inflammation, 140 mg uric acid sodium salt were dissolved in 7 ml distilled water to form a uniform MSU suspension (20 mg/ml). On the first day of the experiment, a single injection of 0.5 ml MSU, with a needle (diameter: 0.45 mm), was administered through an intra-articular injection of MSU inside the knee joint of the right hind limb. Each rat was observed after articular injection to ensure no leakage of the MSU suspension and that it was equally retained after each injection.

### Drug administration

The administrated solutions of BERB and Para were prepared in distilled water. All the rats were received an oral treatment of either BERB or Para 48 h after a single MSU injection in the knee joint, for consecutive 14 days^36^. The oral treatments were received in the morning period between 9:11 a.m. for consecutive 14 days.

### Experimental design

Rats were divided randomly into four groups:

- *Group 1* Negative control group: rats were received no injections or treatments.
- *Group 2* MSU “GA arthritic” positive control group: rats were administrated a single intra-articular injection of 0.5 mL MSU inside the articular cavity of knee joint^36^.
- *Group 3* Arthritis group treated with BERB (MSU + BERB): MSU-induced GA rats received BERB (50 mg/kg of body weight per day, orally) daily for 14 days^31^.
- *Group 4* Arthritis group treated with Para (MSU + Para): MSU-induced GA rats received Para (50 mg/kg of body weight per day, orally).

The treatment started 48 h post-MSU induction, and lasted for 14 days.

### Exclusion criterion

MSU-induced rats that exhibited a weight loss of > 20% were excluded from the current study; however, this was not applicable.

### Assessment of gait score

Gait score was evaluated to assess the behavioral disturbance according to the method of Pan et al.^37^. Gait scores of hind limbs were graded, according to severity, from 1 to 4 as follows: “1 = normal gait; 2 = slightly impaired gait (slight ataxia and foot splay); 3 = moderately impaired gait (obvious ataxia and foot splay with limb abduction during ambulation); 4 = severely impaired gait (inability to support body weight and foot splay).

### Assessment of joint inflammation: measurement of the width of knee and ankle joints

To determine the extent of tissue swelling, as an index of inflammation, the vernier caliper (Made in Germany) was used to measure the knee and ankle joint-width of rats. Ankle-width measurements were done in all groups 48 h (post-MSU induction), while knee-width measurements were done in all groups both 48 h (post-MSU induction) and on 14th day (at the end of the treatment).

#### Blood and sample collection and processing

At the end of the experimental period, Thiopental sodium (50 mg/kg, i.p., Thiopental®, Biochemie GmbH, Vienna, Austria) was used to anesthetize rats that were fasted overnight, with free access to water; the anesthetized rats were sacrificed by decapitation. Blood samples were obtained from the orbital plexus of eyes. The blood was centrifuged at 1200 *g* for 20 min and the serum withdrawn and stored at − 80 °C. Stored samples were used for assaying inflammatory mediators and lipid peroxidation.

#### Histopathological examination of knee and ankle joints

The right hind limb knee and ankle joints were fixed in neutral buffered formalin 10%, washed, decalcified by EDTA, dehydrated, cleared, and embedded in paraffin. The paraffin-embedded blocks were sectioned at 5µ-thickness and stained with Hematoxylin and Eosin^38^; and were examined blindly by a pathologist, unaware of the experiment, using a light microscope (Olympus BX50, Japan).

#### Histopathological lesion scoring of knee and ankle joints

Histopathological alterations were scored as (0, 1, 2, and 3) corresponding to (no, mild, moderate, and severe) change, the grading by percentage as “ < 30%” mild changes, “ < 30% – 50%” moderate changes, and “> 50%” severe changes^39^.

## Biochemical analyses

### Determination of serum levels of lipid peroxidation end-product: malondialdehyde (MDA)

Lipid peroxidation, assessed as serum malondialdehyde (MDA) levels, was evaluated spectrophotometrically according to Ohkawa et al.^40^ at 534 nm; using standard colorimetric diagnostic kits.

### Determination of serum levels of monocyte chemotactic protein 1 (MCP-1), vascular endothelial growth factor (VEGF), and prostaglandin E2 (PGE2)

The chemokine (MCP-1), cytokine (VEGF) and inflammation mediator (PGE2) biomarkers were determined using the ELISA method in the serum samples collected from different experimental groups. All ELISA systems were purchased commercially (MCP-1, VEGF, PGE2 from Endogen, Rockford, USA), and performed following the protocol from the manufacturer. According to the manufacturers’ information, the sensitivity is < 5 pg/ml (ELISA range: MCP-1 is 38–1500 *p*g/ml; VEGF is 23.8–1500 pg/ml and PGE2 is 31.25–2000 *p*g/mL).

### Molecular analyses: expression of elastase, cyclooxygenase-2 (COX-2), matrix metalloproteinase-9 (MMP-9), and myeloperoxidase (MPO) genes

*RNA isolation: RNA isolation:* Blood samples were collected from treated rats with MSU, MSU + BERB, and MSU + Paracetamol. Total RNA was isolated by the standard TRIzol® Reagent extraction method (Cat. no. 15596026, Invitrogen GmbH, Darmstadt, Germany)^41,42^. RNA purity (260/280 nm ratio: 1.8–2.1) and integrity were confirmed by agarose gel electrophoresis. Total RNA was treated with 1 U of RQ1 RNAse-free DNAse (Cat no. / ID. 74,104, QIAGEN, Amtsgericht Düsseldorf, Germany); to digest DNA residues.

*Reverse transcription (RT) reaction:* The complete isolated Poly(A)^+^ RNA was reverse transcribed into cDNA, using RevertAid™ First Strand cDNA Synthesis Kit (Cat. No. K1621, Thermo Fisher Scientific GmbH, Dreieich, Germany). Five µg of total RNA was used with a reaction mixture, termed as master mix. The mixture of each sample was centrifuged for 30 s at 1000 g and transferred to the thermocycler (Biometra GmbH, Göttingen, Germany). The RT reaction was carried out at 25 °C for 10 min, then by 1 h at 42 °C, then 5 min at 99 °C to stop the reaction. Afterwards the reaction tubes containing RT preparations were used for DNA amplification.

*Real Time-Polymerase Chain Reaction (RT-PCR):* StepOne™ Real-Time PCR System from Applied Biosystems (Thermo Fisher Scientific, MA USA) was used to determine rat’s sample copy number. PCR reactions were set up in 25 μL reaction mixtures containing 12.5 μL 1 × SYBR® Premix Ex TaqTM (Cat. No. RR420A, TaKaRa, Biotech. Co. Ltd., Dalian, China), 0.5 μL 0.2 μM sense primer, 0.5 μL 0.2 μM anti-sense primer, 6.5 μL distilled water, and 5 μL of cDNA template^43,44^.

The program was divided into 3 stages: (1st) 3 min at 95 °C, (2nd) 40 cycles in which each one is divided into 3 steps: (a) 15 s at 95 °C; (b) 30 s at 55 °C; and (c) 30 s at 72 °C, (3rd) melting curve phase which started from 60 °C up to 95 °C. The quantitative values of specific genes were normalized on the GAPDH standard gene. GAPDH is a popular housekeeping gene that is often used as a stable marker for constant gene expression. The sequence of primers is listed in Table 1. Relative quantification using 2^−ΔΔCt^ method was utilized to calculate the relative abundance (fold changes) of each gene within each group^45,46^.

**Table 1:** Primers sequence used for *qRT-PCR.*

Gene	Primer sequence	NCBI reference
Elastase	**F:** CCA CAC TCA TTG CCA GGA AC	NM_001106767.1
	**R:** GCG TTA ATG GTA GCT GAG CC	
COX-2	**F:** GCT TAA AGA CCG CAT CGA GG	S67722.1
	**R:** GGC TGA ACT CAC ACA TTG CA	
MMP-9	**F:** AGG ATG GTC TAC TGG CAC AC	NM_031055.2
	**R:** GTG CAG GAC AAA TAG GAG CG	
MPO	**F:** TGG GGA GAA GCT TTA CCA GG	XM_032913217
	**R:** GCG ATT CGA GGG TCT ACT GA	
GAPDH	**F:** GAG ACA GCC GCA TCT TCT TG	NM_017008.4
	**R:** TGA CTG TGC CGT TGA ACT TG	

COX-2, cyclooxygenase-2; MMP-9, matrix metallopeptidase-9, MPO, myeloperoxidase, GAPDH, glyceraldehyde-3-phosphate dehydrogenase.

### DNA fragmentation assay

#### DNA gel electrophoresis laddering assay in knee joints

This method is a qualitative tool for determination the DNA fragmentation for the cell death by detecting DNA fragments using agarose gel electrophoresis. The DNA fragmentation assay was conducted according to the method of Yawata^47^ with some modifications. Briefly, knee cells were homogenized and centrifuged at 800 rpm for 10 min. Approximately 1 × 10^6^ cells of each treatment were plated, harvested, and washed with Dulbecco`s Phosphate Buffered Saline (PBS). Then cells were lysed with the lysis buffer for 30 min on ice. Lysates were vortexed and centrifuged at 10,000 g for 20 min. Fragmented DNA was extracted with an equal volume of neutral phenol: chloroform: isoamyl alcohol mixture (25:24:1) and analyzed electrophoretically on 2% agarose gels containing 0.1 μg /ml ethidium bromide.

#### Diphenylamine reaction procedure

This assay is a quantitative tool for measuring apoptosis by determining the percentage of DNA fragmentation as into oligosomal-sized fragments. Knee cells were collected immediately, and then the cells were lysed in 0.5 ml of lysis buffer, and then centrifuged at 10,000 rpm for 20 min at 4 °C. Then, 0.5 ml of 25% Trichloroacetic acid (TCA) was added to the pellets and the supernatants, and incubated at 4 °C for 24 h. The cells were then centrifuged for 20 min at 10,000 rpm at 4 °C and the pellets were suspended in 80 ml of 5% TCA, followed by incubation at 83 °C for 20 min. Then, 160 ml of Diphenyl Amine (DPA) solution was added to each cell sample and incubated at RT for 24 h^48^. The percentage of fragmented DNA was calculated from absorbance reading at 600 nm wavelengths using the following formula:

$$\begin{aligned}`` \% \,{\text{Fragmented}}\,{\text{DNA}} = & \left[ {{\text{optical}}\,{\text{density}}{\mkern 1mu} \left( {{\text{supernatants}}} \right)/} \right[{\text{optical}}\,{\text{density}}\left( {{\text{supernatants}}} \right) \\ & + {\text{optical}}\,{\text{density}}\,\left( {{\text{pellets}}} \right)]*100 {\text{''}}\\ \end{aligned}$$

### Statistical analysis

The data was tabulated using Statistical Package for Social Science (IBM-SPSS 24 for windows; SPSS Inc., Chicago, USA). Data analysis was conducted using a one-way analysis of variance (ANOVA), followed by Duncan’s post-hoc test. The results were presented as “mean ± standard error of the mean (SEM)”. Statistical significance was defined at the *p*-value < 0.05.

## Results

### Effect of berberine (BERB) on joint edema in MSU crystal-induced GA rats

Edema and swelling of articular joints (ankle and knee) were evaluated 48h post-MSU injection in the knee joint; it was evidenced visually that the hind paw was more swollen, and the knee circumferences were also increased.

**Fig. 1:**
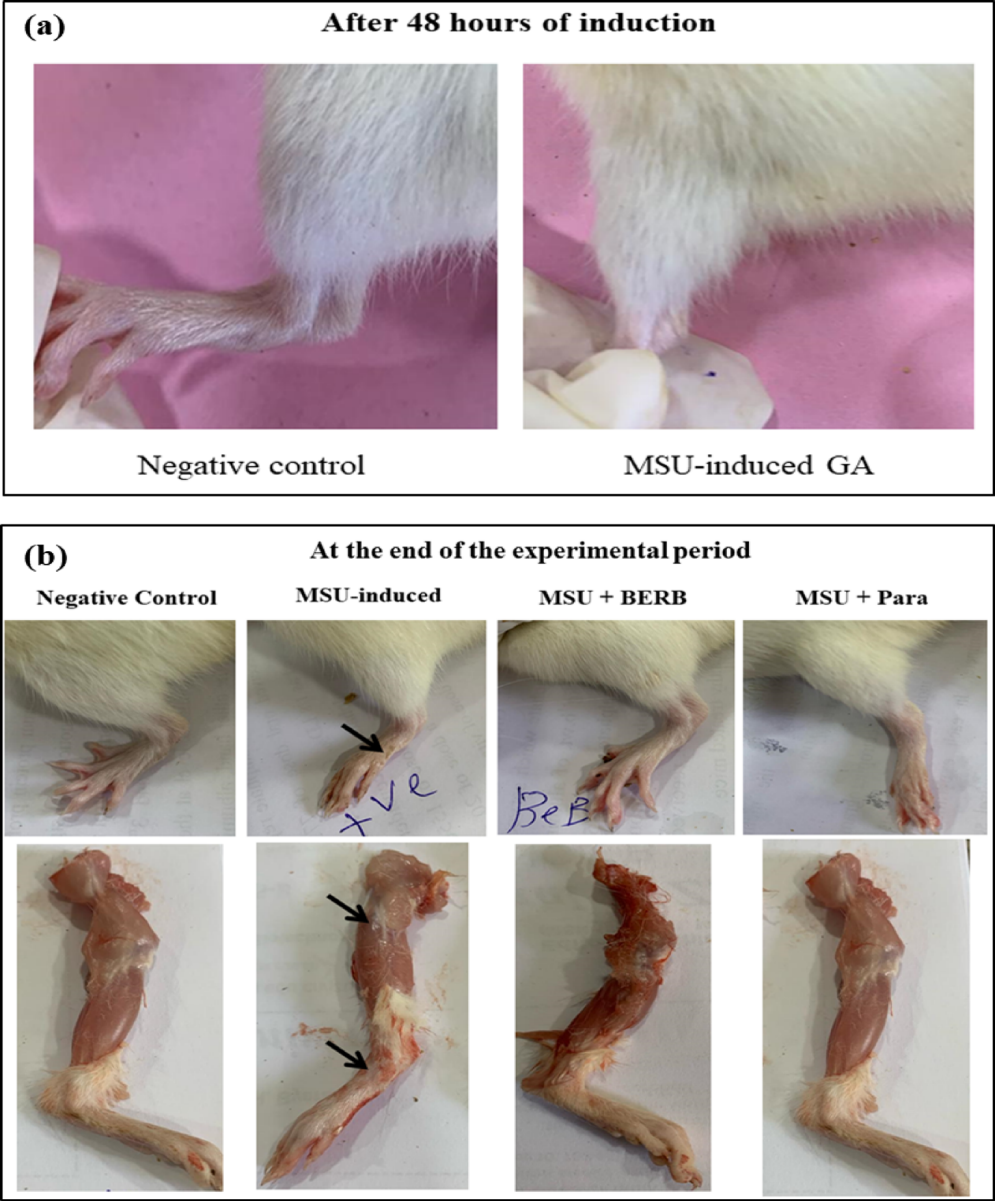
Representative images of the limb from each group showing the knee and ankle (paw) joints of different experimental groups: Negative control, Monosodium Urate (MSU)-induced arthritic rats, treated MSU + Berberine (BERB, 50 mg/kg/day, oral), treated MSU + Paracetamol (Para, 50 mg/kg/day, oral). (**a**) 48 h post MSU-induction, (**b)** At the end of the experimental period (after 14 days).

**Fig. 2:**
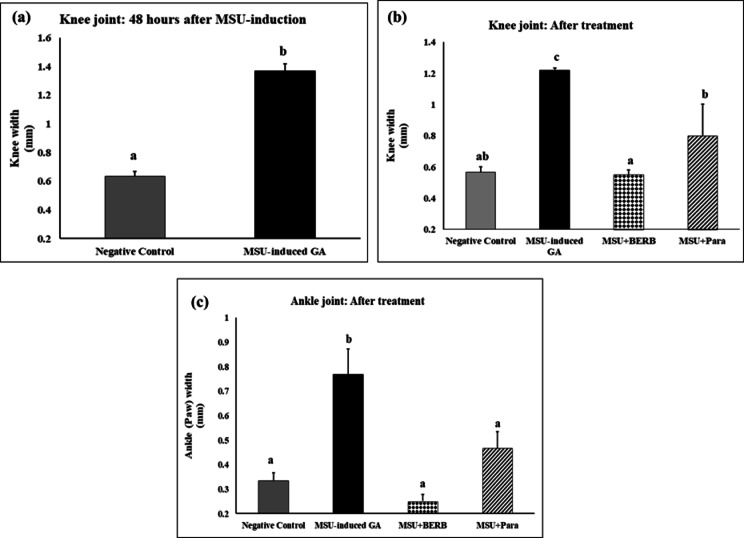
Effect of treatment on MSU-induced oedema in knee and ankle joints in different experimental groups: Negative control, Monosodium Urate (MSU)-induced arthritic rats, treated MSU + Berberine (BERB, 50 mg/kg/day, oral), treated MSU + Paracetamol (Para, 50 mg/kg/day, oral). Results are presented as mean ± SEM. Bars show mean and standard error at the group level with different letters (a, b, c, d) indicating statistical significance at *P* ≤ *0.05*.

Joints (ankle and knee)-width measurements were conducted on 14th day (at the end of the treatment) to evaluate the effect of treatments in the GA-induced rats, the BERB-treated and the Para-treated GA groups, the knee swelling and ankle thickness decreased significantly. These results suggest that BERB and Para treatments mitigate MSU-associated edema and articular inflammation. The results suggested that BERB was more effective than Para in alleviating MSU crystal-induced edema and swelling of articular joints in GA rats (Fig. 1a,b); (Fig. 2a–c).

### Effect of berberine (BERB) on gait quality in MSU crystal-induced GA rats

**Fig. 3:**
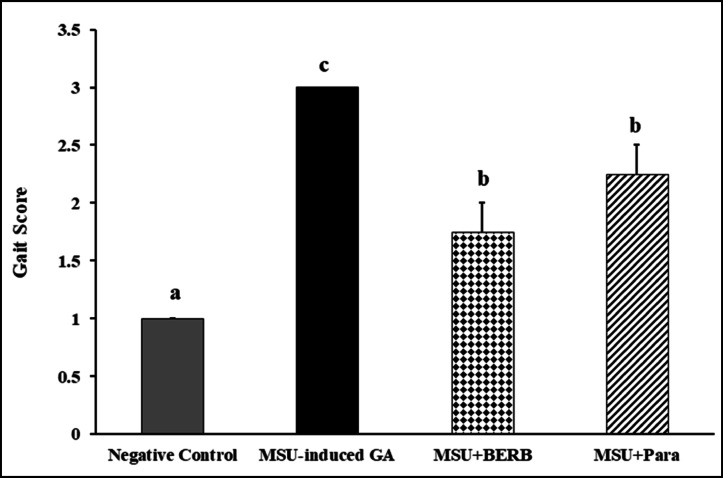
Effect of treatment on MSU-induced gait abnormalities in different experimental groups: Negative control, Monosodium Urate (MSU)-induced arthritic rats, treated MSU + Berberine (BERB, 50 mg/kg/day, oral), treated MSU + Paracetamol (Para, 50 mg/kg/day, oral). Results are presented as mean ± SEM. Bars show mean and standard error at the group level with different letters (a, b, c, d) indicating statistical significance at *P* ≤ *0.05*.

Rats from both control and treated groups were subjected to an evaluation of gait quality by estimating the gait score. Negative control rats showed normal gait throughout the study duration. Nevertheless, the MSU-induced GA rats demonstrated gait abnormalities and ambulation difficulty, represented as swelling and lameness in both knee and ankles. Statistically, a significant increase by nearly threefold (200%) in the gait score was estimated in MSU-induced rats, as compared to the score of control rats (Fig. 3). On the other side, treatment of MSU-induced rats with either BERB or Para significantly lowered the gait score to be 41.67 and 25%, respectively as compared to the score of MSU-induced rats. BERB was more effective than Para in improving gait quality in MSU-induced GA rats.

### Effect of BERB on MSU crystal-induced histopathological changes GA rats: assessed by hematoxylin and eosin (H&E) staining

To investigate the potential of BERB or Para on mitigating synovitis or arthritis, and suppressing inflammation such as infiltration of inflammatory cells “*e.g.* neutrophil” in MSU-induced GA rats, staining with hematoxylin and eosin was conducted in the articular joints of knee and paw.

In addition, the scoring of histopathological changes in the knee/ankle joints of MSU-induced gouty rats and all treated groups is presented in Table 2 that demonstrated that MSU-induced GA rats exhibited the highest score of histopathological alterations in the ankle and the knee joints. In contrast, the treated GA groups with either BERB or Para mitigated those histopathological alterations in the examined joints and accordingly decreased the histopathological scores.

**Table 2:** Scoring of histopathological changes in the knee/ankle joints of MSU-induced gouty rats and all treated groups.

Scoring of histopathological alterations in the knee joint of all treated groups				
**Lesions**	**Negative control**	**MSU-induced GA**	**MSU + BERB**	**MSU + Para**
Irregularity and deformities of articular surface	0	3	0	1
Necrosis of chondrocytes	0	3	1	2
Synovial membrane edema	0	3	2	0
Bone destruction	0	3	0	0

Scoring of histopathological alterations in the ankle joint of all treated groups				
**Lesions**	**Negative control**	**MSU-induced GA**	**MSU + BERB**	**MSU + Para**
Irregularity and deformities of articular surface	0	3	0	1
Necrosis of chondrocytes	0	3	1	1
Synovial membrane edema	0	2	1	1
subcutaneous tissue infiltration with inflammatory cells	0	2	1	0

The scoring system of histopathological changes in the knee/ankle joints is “**0:** no change, **1:** mild change, **2:** moderate, **3:** severe” histopathological change.

**Fig. 4:**
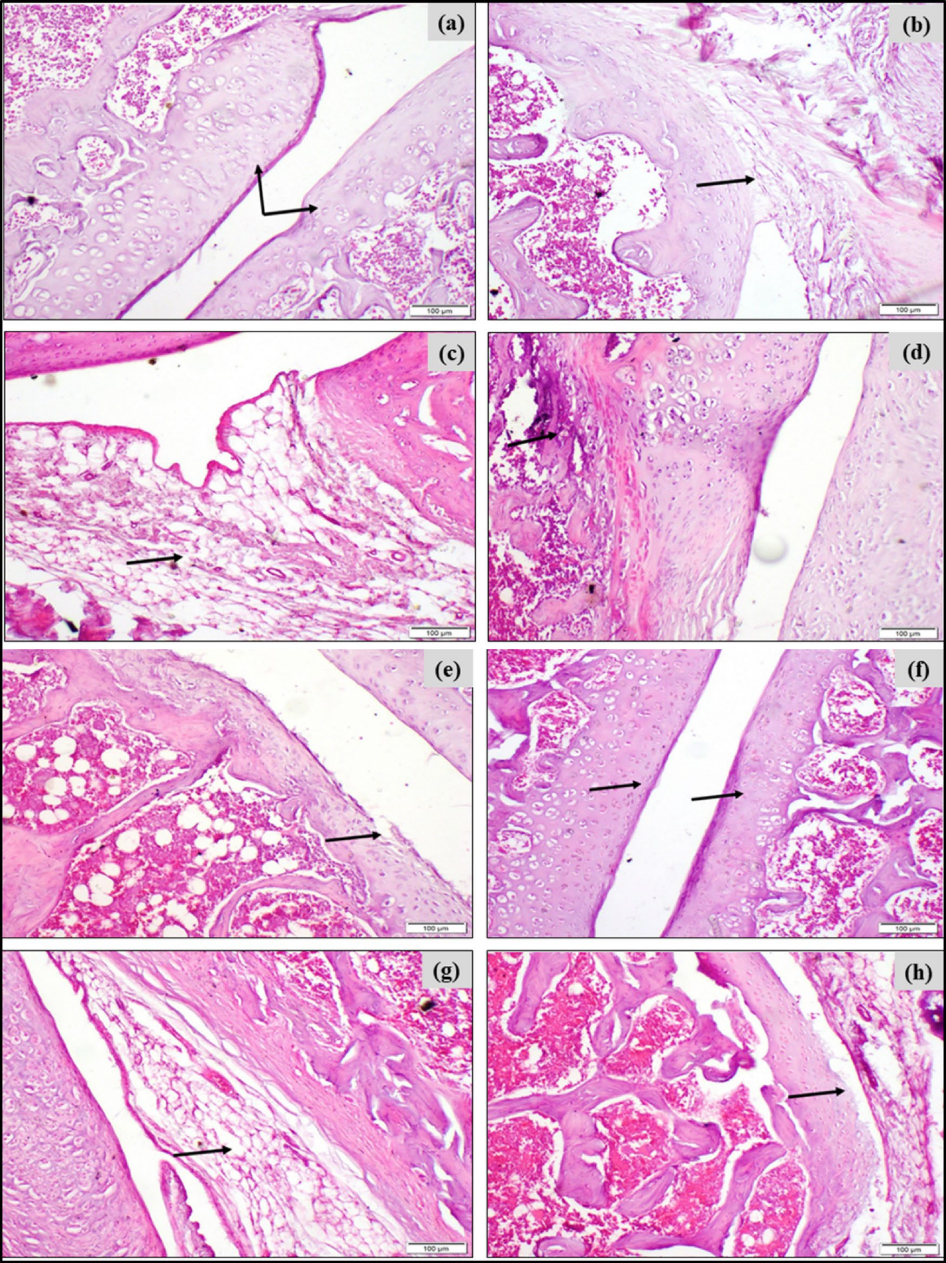
Effect of treatment on MSU-induced pathological changes in knee joints in different experimental groups: (**a**) Photomicrograph, articular surface showing normal histological structure (arrows) of negative control group (H&E, scale bar 100 µm). (**b**) Photomicrograph, synovial membrane showing normal histological structure (arrow) of negative control group (H&E, scale bar 100 µm). (**c**) Photomicrograph, MSU-induced knee showing edema of synovial membrane (arrow) (H&E, scale bar 100 µm). (**d**) Photomicrograph, MSU-induced knee showing destruction of bone (arrow) (H&E, scale bar 100 µm). (**e**) Photomicrograph, MSU-induced knee showing irregular articular surface and necrosis of chondrocytes (arrow) (H&E, scale bar 100 µm). (**f**) Photomicrograph, MSU-induced knee treated with BERB (50 mg/kg/day) showing smooth articular surface (arrows) (H&E, scale bar 100 µm). (**g**) Photomicrograph, MSU-induced knee treated with BERB (50 mg/kg/day) showing moderate synovial membrane edema (arrow) (H&E, scale bar 100 µm). (**h**) Photomicrograph, MSU-induced knee treated with Para (50 mg/kg/day) showing mildly destructed articular surface (arrow) (H&E, scale bar 100 µm).

Negative control group exhibited an intact knee joint tissue structure, without infiltration of inflammatory cells (Figs. 4a,b). In the model MSU-induced GA group, the knee joint tissue demonstrated an obvious damage and loose tissue structure, synovitis with some necrotic regions, the cells showed oedema and focal hemorrhage, and there was infiltration of inflammatory cells (Figs. 4c,d,e). In the treated GA group with either BERB (Fig. 4f,g) or Para (Fig. 4h), the joint tissue demonstrated a nearly intact joint structure, and cells were regularly distributed. The infiltration of neutrophils “inflammatory cells” was mainly in the outer layer.

**Fig. 5:**
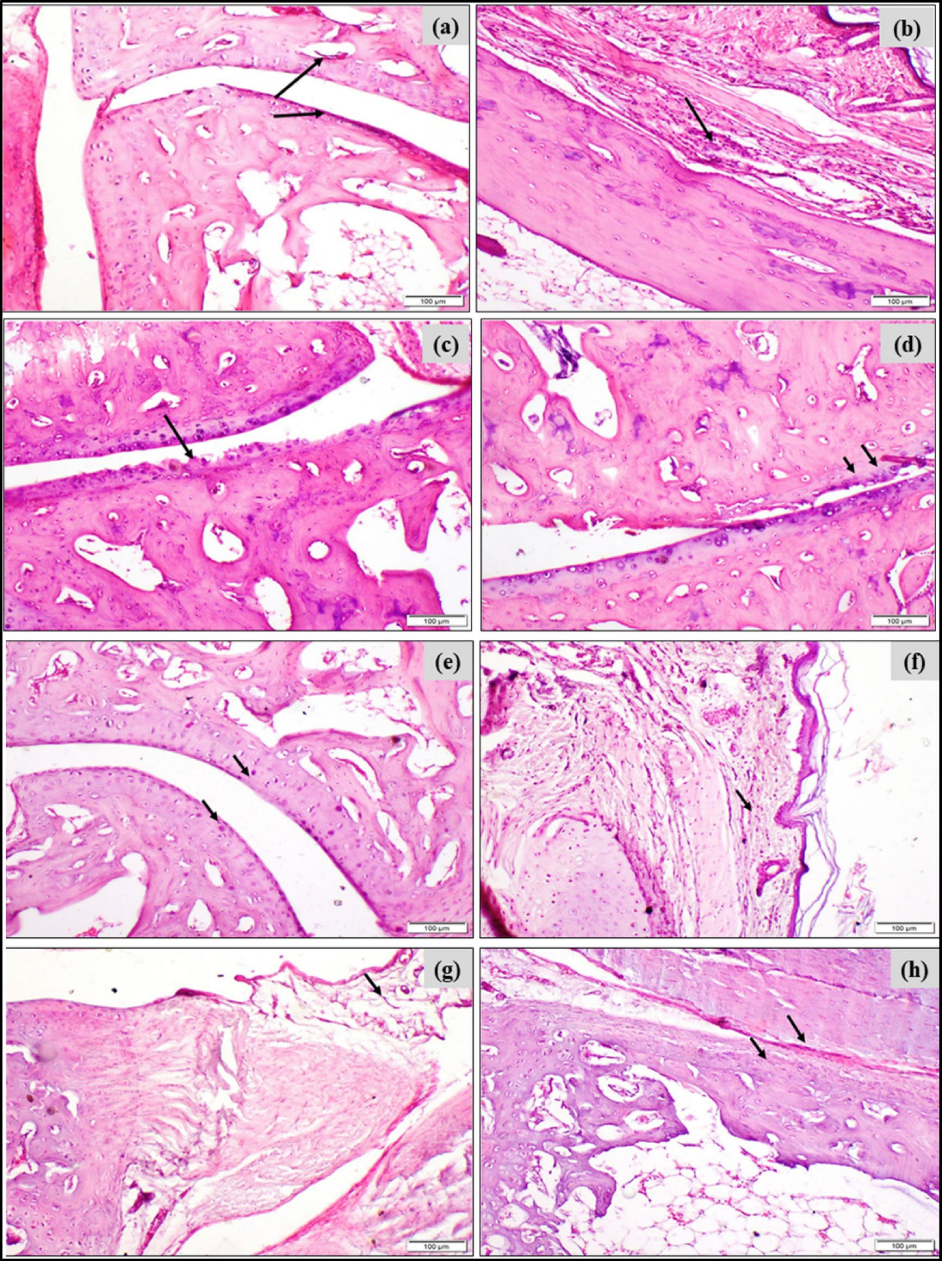
Effect of treatment on MSU-induced pathological changes in paw (ankle) joints in different experimental groups: (**a**) Photomicrograph, articular surface of negative control group showing normal histological structure (arrows) (H&E, scale bar 100 µm). (**b**) Photomicrograph, subcutaneous tissue showing infiltration of mononuclear inflammatory cells (arrow) in MSU-induced paw (H&E, scale bar 100 µm). (**c**) Photomicrograph, rat paw showing irregularity of articular surface (arrow) in MSU-induced paws (H&E, scale bar 100 µm). (**d**) Photomicrograph, articular surface showing necrosis of chondrocytes (arrows) in MSU-induced paws (H&E, scale bar 100 µm). (**e**) Photomicrograph, MSU-induced paw treated with BERB (50 mg/kg/day) showing smooth articular surface (arrows) (H&E, scale bar 100 µm). (**f**) Photomicrograph, MSU-induced paw skin treated with BERB (50 mg/kg/day) showing mild infiltration of mononuclear inflammatory cells (arrow) (H&E, scale bar 100 µm). (**g**) Photomicrograph, MSU-induced paw treated with Para (50 mg/kg/day) showing mild edema of synovial membrane (arrow) (H&E, scale bar 100 µm). (**h**) Photomicrograph, MSU-induced paw treated with Para (50 mg/kg/day) showing smooth articular surface (arrows) (H&E, scale bar 100 µm).

Negative control group exhibited an intact ankle joint tissue structure, without recruitment of inflammatory cells (Fig. 5a). On the other hand, H&E-stained tissue in the ankle joint demonstrated obvious synovitis pattern and infiltration of inflammatory leucocytes, with vascular thrombosis and tissue necrosis in the MSU-induced GA group (Fig. 5b,c,d). However, synovitis and acute inflammation were attenuated in the BERB- and Para-MSU-treated groups, and demonstrated mild infiltration of scattered leucocytes, as compared to that of the MSU injected group (Figs. 5c,d).

We could propose that BERB and Para demonstrated a similar healing impact in ameliorating MSU-induced histopathological changes in the articular joints of GA rats.

### Effect of BERB on lipid peroxidation in MSU crystal-induced GA rats

**Fig. 6:**
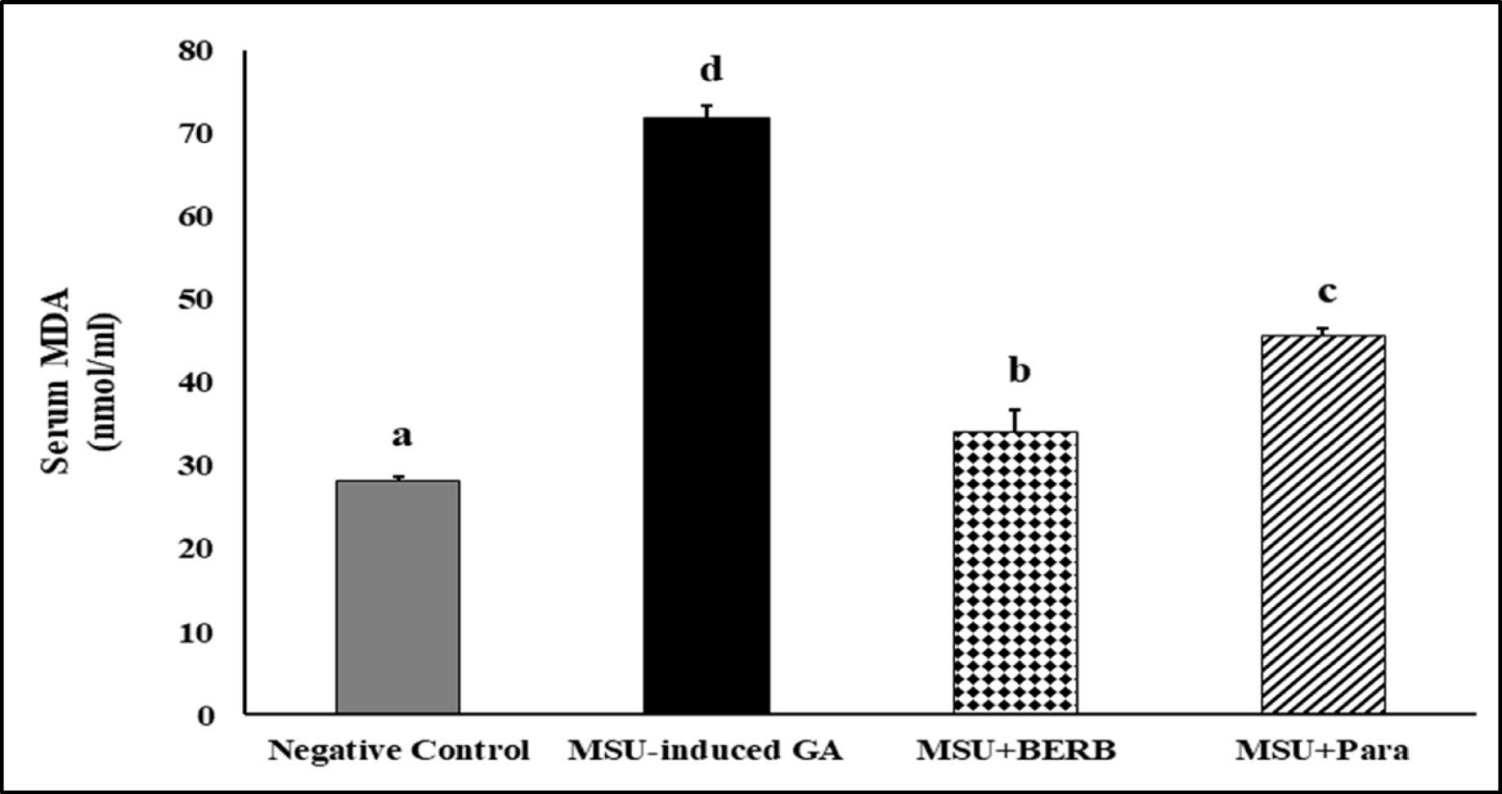
Effect of treatment on MSU-induced lipid peroxidation in different experimental groups: Negative control, Monosodium Urate (MSU)-induced arthritic rats, treated MSU + Berberine (BERB, 50 mg/kg/day, oral), treated MSU + Paracetamol (Para, 50 mg/kg/day, oral). Results are presented as mean ± SEM. Bars show mean and standard error at the group level with different letters (a, b, c, d) indicating statistical significance at *P* ≤ *0.05*. MDA: Malondialdehyde.

Figure 6 showed the anti-oxidant effects of BERB and Para on serum MDA in different experimental groups. Serum MDA levels were elevated significantly in MSU-induced arthritic rats (154.75%), as compared with the control rats. However, treatment of MSU-induced arthritic rats with BERB and Para reduced MDA levels to 52.32, 36.29% respectively, as compared to MSU-rats. The results suggested that BERB exhibited higher anti-oxidative potential than Para against MSU-induced oxidative stress in GA rats.

### Effect of BERB on serum levels chemokine (MCP-1), cytokine (VEGF) and inflammation mediator (PGE2) in MSU crystal-induced GA rats

**Fig. 7:**
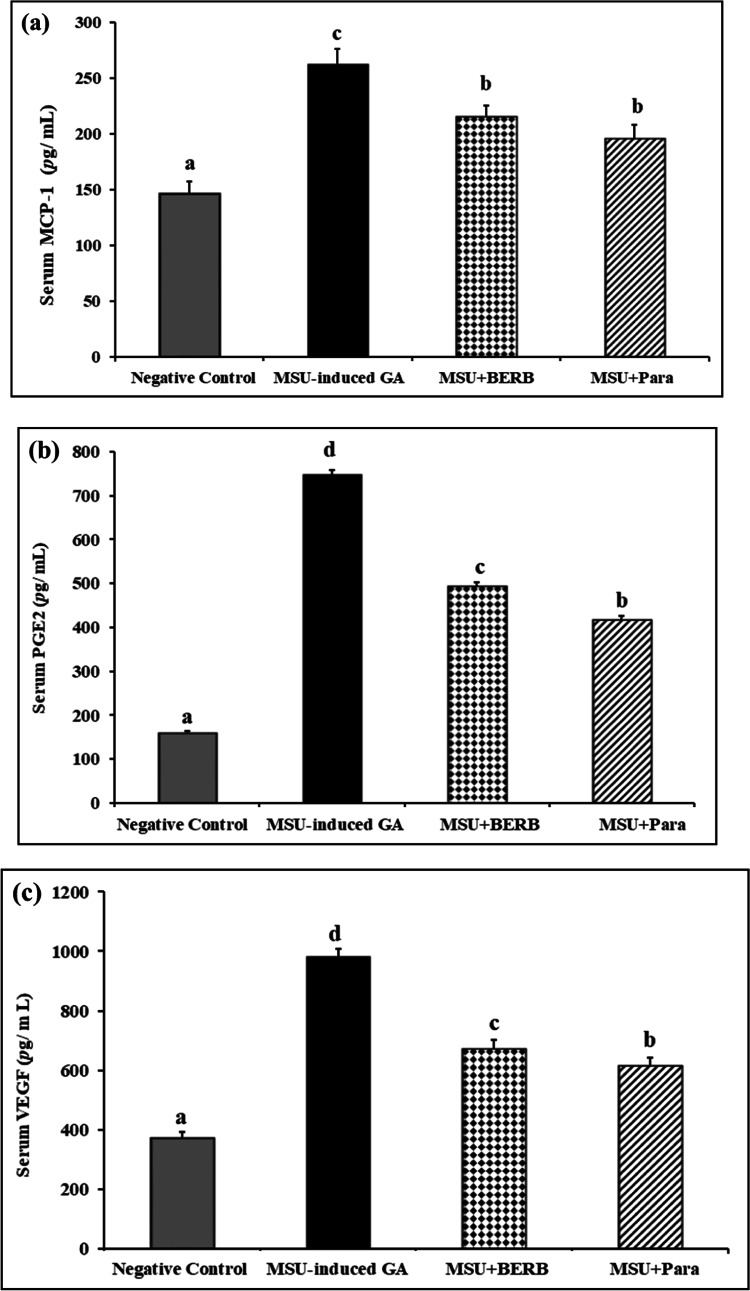
Effect of treatment on the MSU-induced alterations in the serum levels of MCP-1, VEGF, PGE2 of different experimental groups: Negative control, Monosodium Urate (MSU)-induced arthritic rats, treated MSU + Berberine (BERB, 50 mg/kg/day, oral), treated MSU + Paracetamol (Para, 50 mg/kg/day, oral). Results are presented as mean ± SEM. Bars show mean and standard error at the group level with different letters (a, b, c, d) indicating statistical significance at *P* ≤ *0.05*. (**a**) Monocyte chemotactic protein 1 (MCP-1), (**b**) Vascular endothelial growth factor (VEGF), and (**c**) Prostaglandin E2 (PGE2).

The chemokine (MCP-1), cytokine (VEGF) and inflammation mediator (PGE2) biomarkers were estimated in serum of different experimental groups, as presented in Fig. 7. The results showed that the levels of MCP-1, VEGF and PGE2 were increased significantly (*P* < 0.001) in GA rats, as compared with those in control rats. The levels of MCP-1, VEGF and PGE2 were reached to 179.1%, 264.7% and 469.6%, respectively in GA rats, as compared with those in control rats.

On the other side, treatment of GA rats with BERB reduced significantly (*P* < 0.05) the levels of MCP-1, VEGF and PGE2, as compared with those in GA rats; BERB treatment reduced MCP-1, VEGF and PGE2 levels by 17.7%, 31.5% and 34.0%, respectively in comparison with those in GA rats. In the same line, treatment of GA rats with Para reduced significantly (*P* < 0.05), MCP-1, VEGF and PGE2 levels, as compared with those in GA rats. on the other side, Para treatment decreased the serum levels of MCP-1, VEGF, and PGE2 by 25.5%, 37.3%, and 44.1% respectively, in comparison with those in GA rats (Fig. 7a,c). The results proposed that BERB and Para exhibited comparable anti-inflammatory activities against MSU-induced inflammation in GA-induced rats.

### Effect of berberine (BERB) on the expression of elastase and inflammatory mediators (COX-2, MMP-9 and MPO) in MSU crystal-induced GA rats

**Fig. 8:**
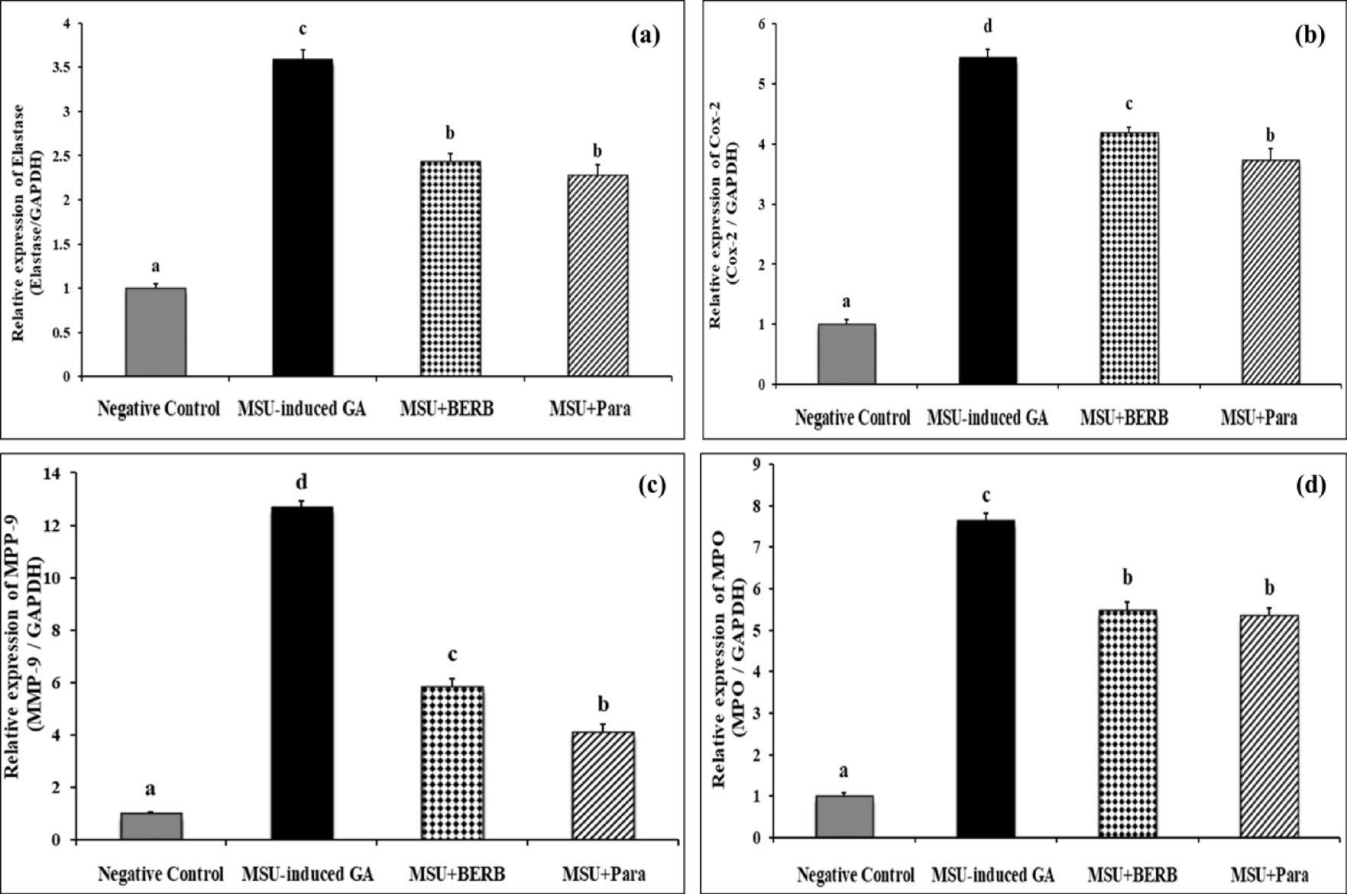
Effect of treatment on the MSU-induced alterations in the expression of Elastase, COX-2, MMP-9, and MPO genes in blood samples of different experimental groups: Negative control, Monosodium Urate (MSU)-induced arthritic rats, treated MSU + Berberine (BERB, 50 mg/kg/day, oral), treated MSU + Paracetamol (Para, 50 mg/kg/day, oral). Glyceraldehyde-3-Phosphate Dehydrogenase (GAPDH) was specified as the internal control gene. Results are presented as mean ± SEM. Bars show mean and standard error at the group level with different letters (a, b, c, d) indicating statistical significance at *P* ≤ *0.05*. (**a**) Elastase, (**b**) Cyclooxygenase-2 (COX-2), (**c**) Matrix metalloproteinase-9 (MMP-9), and (**d**) Myeloperoxidase (MPO).

This study investigated the expression analysis of Elastase, COX-2, MMP-9 and MPO genes in serum samples of gouty rats exposed to MSU crystals and treated with either BERB or Para (Figs. 8a,d). The results revealed significantly up-regulated the expression (*P* < 0.001) levels of Elastase, COX-2, MMP-9, and MPO in MSU-induced arthritic rats; by 359%, 543%, 1268% and 764%, respectively, as compared to those in control rats.

In contrast, treatment of MSU-induced arthritic rats with BERB (*P* < 0.01) significantly down-regulated the expression levels of Elastase, COX-2, MMP-9 and MPO genes by 32.3, 23, 53.9, and 28.3%, respectively as compared with those in arthritic rats. Additionally, treatment of arthritic rats with Para inhibited significantly (*P* < 0.01) the expression level of Elastase, COX-2, MMP-9, and MPO genes by 36.5, 31.3, 67.5, and 29.8%, respectively as compared with those in arthritic rats. Regarding the molecular expression of elastase and inflammatory mediators (COX-2, MMP-9 and MPO); it could be suggested that BERB and Para demonstrated almost similar anti-inflammatory potential, against MSU-induced inflammation in GA rats.

### Effect of berberine (BERB) on DNA fragmentation in knee tissues of monosodium urate (MSU) crystal-induced GA rats and different experimental groups

**Fig. 9:**
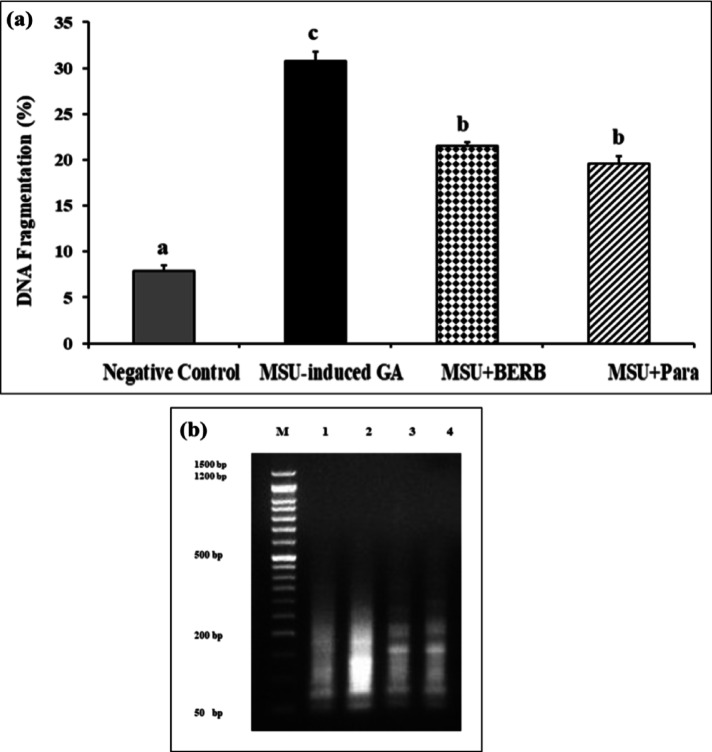
Effect of treatment on DNA fragmentation detected in knee cells of rats of different experimental groups: Negative control, Monosodium Urate (MSU)-induced arthritic rats, treated MSU + Berberine (BERB, 50 mg/kg/day, oral), treated MSU + Paracetamol (Para, 50 mg/kg/day, oral). (**a**) Results, as quantitative values of DNA fragmentation, are presented as mean ± SEM. Bars show mean and standard error at the group level with different letters (a, b, c, d) indicating statistical significance at *P* ≤ *0.05*. (**b**) Qualitative values of DNA fragmentation in knee cells of arthritis rats. Lane M: DNA marker; Lane 1: Negative control rats, Lane 2: MSU-induced arthritic rats, Lane 3: treated MSU + BERB rats, and Lane 4: treated MSU + Para rats.

Measurement of DNA fragments using diphenylamine (DPA) assay was used to assess quantitative values of DNA fragmentation rates in knee tissues samples exposed to MSU, BERB, and Para (Table 3, Fig. 9a). However, the **DNA gel electrophoresis laddering assay** was used to assess the qualitative of the DNA fragmentation (Fig. 9b). The results proved that knee samples of control rats exhibited a significant (*P* < 0.01) decrease (7.9 ± 0.56) in DNA fragmentation rates, as compared with those in treated samples. However, the DNA fragmentation results were elevated (30.8 ± 0.93) significantly (*P* < 0.01) in MSU-exposed knee samples as compared with control, MSU + BERB and MSU + Para. The DNA fragmentation values were decreased in knee samples treated with MSU + BERB (19.6 ± 0.45) and MSU + Para (21.5 ± 0.76) compared with those exposed to MSU (30.8 ± 0.93). The results indicated that BERB and Para exhibited comparable anti-apoptotic potential against MSU-induced apoptosis or DNA fragmentation in GA rats.

**Table 3:** DNA fragmentation detected in knee tissues of different treatment groups.

Treatment	DNA Fragmentation %	Change	Inhibition %
Negative control	7.9 ± 0.56^c^	0	0.0
MSU arthritic rats	30.8 ± 0.93^a^	22.9	0.0
MSU + BERB	19.6 ± 0.45^b^	11.7	48.91
MSU + Para	21.5 ± 0.76^b^	13.6	40.61

Groups: Negative control, Monosodium Urate (MSU)-induced arthritic rats, treated MSU + Berberine (BERB, 50 mg/kg/day, oral), treated MSU + Paracetamol (Para, 50 mg/kg/day, oral). Results are presented as mean ± SEM. Means with different superscripts (a, b, c) between treatments in the same column are significantly different at* P* < 0.05.

## Discussion

Elevation of serum uric acid levels in GA patients causes precipitation of uric acid salts and formation of crystals that deposit in the synovial tissues^16^. Similarly, “intra-articular injection of MSU crystals” simulates GA in rodents and generates a painful inflammatory response, similar to spontaneous acute gouty episodes. In the first stages of human MSU-induced GA, polymorphonuclear lymphocytes invade the articular cavity, associated with hyperplasia and hypertrophy of the synovial lining^49^.

MSU crystals, as an endogenous adjuvant, are the most potent pro-inflammatory stimuli that can boost a robust inflammatory response^50^. MSU-induced arthritic rats developed similar alterations to humane arthritis, with respect to induction of inflammatory lesions and oedema following MSU-injection in the articular joint; therefore this GA model is useful for exploring the underlying mechanisms of acute arthritic joint inflammation. In this study, we used MSU-induced arthritic rats as a rodent model of human GA, to investigate the anti-GA mechanisms of BERB.

The swelling of the hind limb occurred gradually, and reached its peak after 48 h after MSU injection (Fig. 2a), and was accompanied with a marked increment of gait score and histological scores in the articular joints (Figs. 3, 4, 5); and caused the joint dysfunction and pathological alterations that impaired the joint movement. Significant swelling of the hind limb including the ankle joints represents severe inflammatory response. Our clinical results run in agreement with Yang et al.^51^. Biochemically, lipid peroxidation was significantly elevated in MSU-induced GA rats, as compared with negative controls; our results run in agreement with Huang et al.^52^ and Elmaidomy et al.^53^ that showed that MSU administration generated oxidative stress.

The main histopathological feature of GA is infiltration (influx) of inflammatory cells such as neutrophils, monocytes, and macrophages into the joint fluid and synovial membrane of joints; then the neutrophils actively engulf “phagocytose” MSU crystals and induce oxidative stress, synovitis, membrane disruption, acute inflammation, and subsequent release of inflammatory mediators, and monocyte chemotactic factor that exaggerate the inflammatory response^51–54^.

The cytokines exert their inflammatory potential in GA through their sustained release or through their ability to inhibit the production of anti-inflammatory cytokines^55^; for example IL-1β and TNF-α are “pleiotropic cytokines” that have been implicated in the pathological mechanism of destruction of both bone and cartilage and have been estimated in rheumatoid synovial fluid and serum^8,16^. Our results demonstrated that the mRNA levels of COX-2, MMP-9, MPO, and elastase were upregulated in the MSU-induced GA rats; our results are in accordance with Goo et al.^8^, Huang et al.^52^, Elmaidomy et al.^53^, and Chen et al.^54^. MSU acts as a stimulator for the inducible COX-2 “an inducible enzyme involved in inflammatory responses” and MMP-9 “an osteoclast-related protein with significant roles in inflammatory responses” in induced GA rats^56–60^. Also, the inflammatory response activates MPO to catalyze the conversion of hydrogen peroxide (H_2_O_2_) to reactive oxygen species (ROS) and hypochlorous acid further harming the articular tissues^61^.

Chemokines such as MMP-9 contribute to the inflammation associated with GA^62^. TNF-α-coordinated MMP-9 causes “matrix degradation” in GA tophi, which mediates the recruitment of inflammatory cells in the inflamed joints^63^. In addition, MMP-9 may worsen arthritis through inducing inflammatory mediators or disrupting the extracellular matrix covering the joints^64^. The overexpression of MMP-9 has been clinically observed in the synovial fluid of arthritic patients; therefore MMP-9 could be used as biomarker for GA; MMP9 activation in synovial fluid samples can reveal the inflammation of the knee joint in GA^65^. Therefore, developing MMP inhibitor can prevent the destruction of the joints^59^.

Similarly, Pouliot et al.^66^ demonstrated that MSU crystals stimulated PGE2 production and COX-2 expression in monocytes. “COX” is a group of inflammatory enzymes that produce prostaglandins including PGE2; the inducible COX-2 form is upregulated in inflamed tissues and is responsible for elevated PGE2 production^67^. PGE2 as one of the prostanoids, which are related to inflammation and osteoclastic activity, may mediate the articular inflammation, cartilage degradation, and angiogenesis resulting in severe pain around the articular joint^68^.

Furthermore, MSU can act as a stimulator for the production of pro-inflammatory mediators through inducing IκBα degradation and activating NF-κB^51,53^. The expression levels of COX-2 and PGE2 are regulated by NF-κB signaling^69^. Under inflammatory state, COX-2 expression is influenced by inflammatory cytokines, causing excessive PGE2 production^70^. Since excessive nitric oxide (NO) and PGE2 production amplify inflammatory reactions, inhibition of COX-2 and inducible nitric oxide synthase (iNOS), which stimulates PGE2 and NO production, is one of the main targets for suppressing inflammation^71^.

In addition, our results showed that the serum MCP-1 and VEGF levels were increased in MSU-induced rats, which runs in agreement with Goo et al.^8^ and Accart et al.^72^. During a gout flare, the flow of neutrophils and macrophages is regulated by cytokines/chemokines, including MCP-1, that coordinate neutrophil recruitment in the inflammatory response^73^. Within the inflamed synovial tissue in RA and OA joint disorder, hypoxia is associated with angiogenesis and the production of new blood vessels that is mediated by the production of VEGF^74^.

Based upon the observed alterations in joint-width “circumference” and histopathological inflammatory lesions post-MSU injection; we examined whether changes in inflammatory markers were attenuated by the treatment with either BERB or Para. BERB or Para treatment showed significant downregulation in Elastase levels; depicting the reduction in neutrophil infiltration. Our results run in agreement with Dinesh and Rasool^75^. The expression levels of these inflammatory mediators were downregulated; indicating that BERB or Para ameliorated gait score, and reduced the serum MDA levels in MSU-induced GA rats. Histopathologically, treatment of MSU-rats with either BERB or Para significantly inhibited the infiltration of neutrophils, as compared with the MSU-induced rats; indicating the anti-GA potentials of BERB or Para to suppress MSU-associated synovitis.

The anti-inflammatory and anti-oxidative activities of BERB or Para on inflammatory mediators and lipid peroxidation contributed to the mitigation of the acute pain in the joint of MSU-induced rats. These anti-arthritic activities of BERB and Para treatment correlated incisively with the alterations in joint-width and histological features.

The GA-inhibitory potential of BERB was almost similar to or better than that of Para, a standard drug for acute GA. Histopathologically, BERB significantly reduced the thickness of the synovial lining and the infiltration influx in GA-induced rats. Thus, BERB may serve as a preliminary clue for ameliorating MSU crystals-induced inflammation and synovitis.

BERB exhibits the potential to mitigate the level and function of PGE2 by inhibiting the “PLA2-COX-2-PGE2-EP2” pathway with the aid of gut microbiota, thereby attenuating inflammation^76^. Furthermore, treatment of MSU-rats with BERB inhibited elevation of MPO activity through deactivation of JNK signaling pathways^61,75,77^.

DNA fragmentation is the main feature of apoptosis, and thus it is used as an indicator of apoptosis or cell death^78^. Herein, it was demonstrated that DNA fragmentation was significantly increased in the knee joints of MSU arthritic rats, on the other side; treatment of MSU arthritic rats with either BERB or Para demonstrated a significant decrement in the knee joints of treated rats, as compared with that from MSU-induced GA rats.

Our results run in agreement with Hwang et al.^79^ that showed that MSU crystals provoked DNA fragmentation in chondrocytes through “NETosis” pathways; “NETosis” is a cell-death pathway that differs from other cell death pathways like apoptosis and necroptosis^79,80^. MSU crystals are involved in the formation of neutrophil extracellular traps (NETs), which are composed of “DNA, histones, granular enzymes, and anti-microbial proteins”. The deposited structures that form in MSU-stimulated neutrophil cultures are similar to tophi, which are the key players of joint destruction in GA^81^; therefore, the aggregation of NET may represent early “tophus formation” and serve to control acute inflammation by containing MSU crystals^82^. Furthermore, the neutrophils could provide a signal stimulating IL-1β release and inflammation. In addition, pyroptosis is implicated in MSU-induced inflammation in the damaged articular joints through stimulating cells to release molecules including DNA, and IL-18 and IL-1β^82,83^.

During an acute gout episode, NET release relies more on the amount of MSU crystals rather than the number of infiltrating leukocytes^84^. Furthermore, a recent in vitro study^85^ showed that the surge in inflammatory responses is ascribed to the potential of small DNA fragments to bind to pattern recognition receptors on Differentiated HL-60 cells (dHL-60) to induce acute inflammation and formation of “dHL-60 NET-MSU aggregates” in the early phase. Therefore, this in vivo study aimed at better confirm the negative impact of MSU crystals on DNA fragmentation and its role in triggering inflammation.

Regarding the choice of Para; to compare its anti-GA activity with BERB; Para is potentially a suitable analgesic, since it exhibits less anti-inflammatory activity than NSAIDs and COX-2 inhibitors^86^. Para, at 50 mg/kg, significantly decreased nociceptive and spontaneous spinal discharges in adjuvant arthritis^87^. It also mitigated inflammatory hyperalgesia without affecting carrageenan inflammation and central hyperalgesia^88^. For more clarification; Bianchi and Panerai^88^ investigated the impact of 3 oral doses of Para (25, 50 and 100 mg/kg) on hyperalgesia and nociception in rats and showed that Para (at doses of 50 and 100 mg/kg) can mitigate central and peripheral hyperalgesia and trigger nociceptive thresholds to a mechanical stimulus in the non-inflamed paws; the authors proposed that Para can alleviate hyperalgesia without impacting nociception and inflammation. Furthermore, Garrone et al.^89^ demonstrated that Para (75 mg/kg and 150 mg/kg; i.p.) could have neuroprotective potential; through halting the development of Post-operative cognitive dysfunction (POCD); in other words, Para-administrated middle-aged rats (75 mg/kg or 150 mg/kg; i.p.) were protected from POCD; suggesting the potential clinical use of Para as first-choice analgesic in POCD, as an alternative to opioids. In addition, Chen et al.^90^ concluded that Para can both influence emotion processing and alleviate pain clinically. They found that the low dose of Para (50 mg/kg) inhibited mechanical pain hypersensitivity in spared nerve injury (SNI)-induced rats, without influencing pain behavior in sham-operated rats, and suggested that a high Para dose (300 mg/kg) increases anxiety-like and anhedonic behavior, and negatively impact recognition memory in sham controls, while in neuropathy, a low Para dose (50 mg/kg) decreases nerve injury-associated anxiety potentially through mitigating neuropathic pain.

Previous experimental studies demonstrated health-promoting activities of BERB; such as regulating metabolic disruptions and exhibiting cardioprotective, nephroprotective, neuroprotective, and anti-diabetic potentials, through exerting anti-inflammatory, anti-oxidative, anti-apoptotic, and anti-cancer activities^91–96^.

The acute toxicity of a certain compound, *e.g.* BERB, correlates with its post-administration blood levels and with the route of administration. Thus, Zuo et al.^97^ proposed that the intestinal absorption of BERB in the animal’s system has its internal limit; any extra BERB will be excreted. In addition, Kheir et al.^98^ analyzed blood BERB content after several administrations and concluded that both the blood BERB concentration and the routes of administration are the main factors that affect the evaluation of acute toxicity of BERB. Moreover, several other studies investigated the toxicology potential of BERB as indicated by the review article of Rad et al.^99^. Jiang et al.^100^ found that oral dose of 100 mg/kg BERB was well-tolerated by rats, and BERB administration was not associated with any toxic impact on the hepatorenal system in rats received 50 mg/kg BERB. Another study by Zhou et al.^101^ showed that BERB administration at doses higher than 50, 100 and 150 mg/kg, after 16 weeks, stimulates hepatic injury in diabetic rats but not in control rats. In rats, the developmental toxicity of BERB has been reported, as the no-observed-adverse-effect level (NOAEL), was 1000 mg/kg/day^102^.

**Fig. 10:**
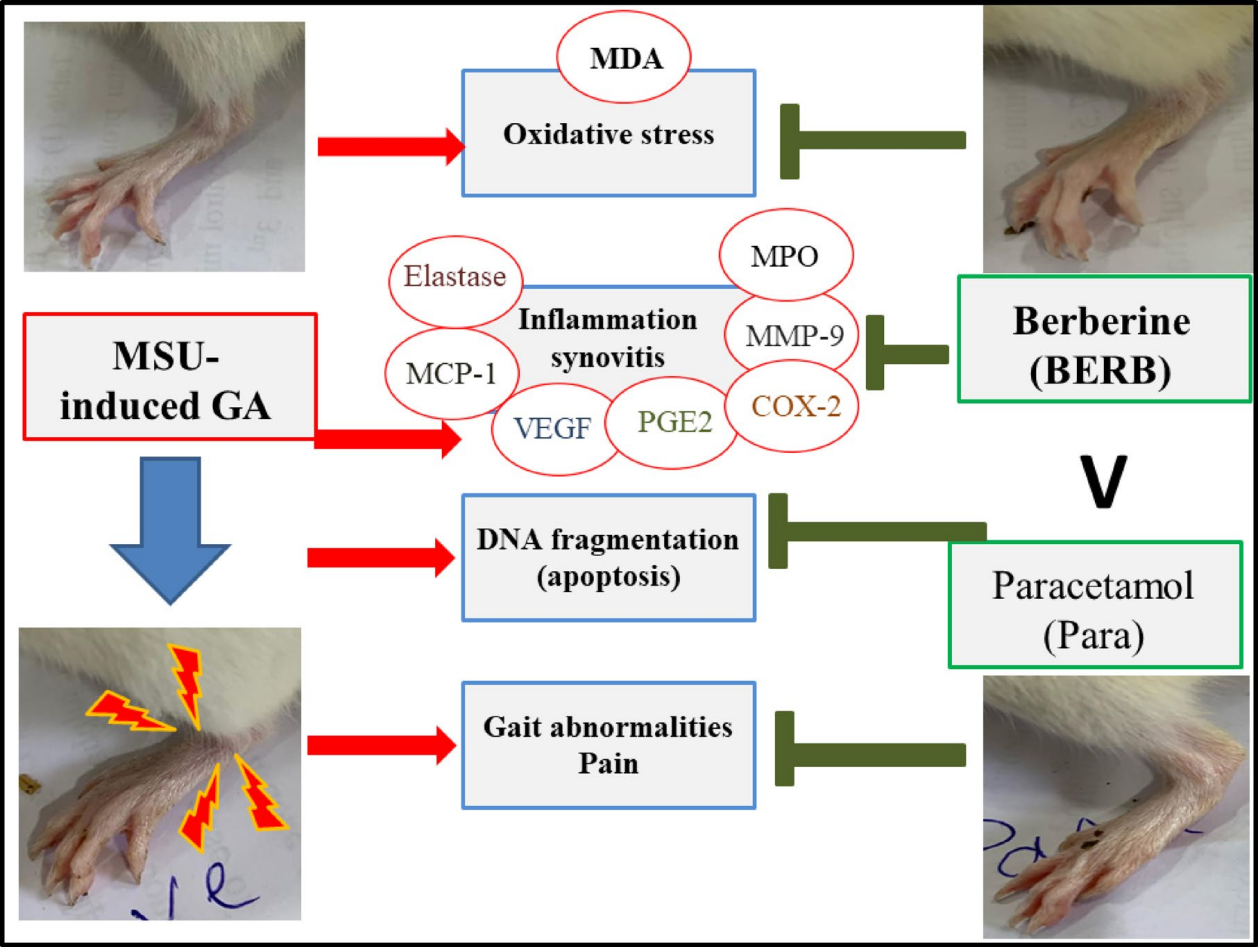
Representative diagram demonstrating the anti-arthritic potential of Berberine (BERB) and Paracetamol (Para) against Monosodium Urate (MSU)-induced gouty arthritis in rats: The anti-arthritic potential of either BERB (50 mg/kg/day, oral) or Para (50 mg/kg/day, oral) is mediated by exerting antioxidative, anti-inflammatory, and anti-apoptotic activities that resulted in improvement of gait quality. However, BERB showed higher anti-arthritic potential than that of Para.

The inflammation and proliferation of synovial cells were demonstrated in the MSU-induced GA model rats. Joint inflammation can exaggerate oxidative damage in the tissue environment, and based on the observed anti-inflammatory, anti-oxidant, and anti- NETosis activities of BERB and Para, it was possible that both treatments also exhibited anti-arthritic activities. In our study, we showed that joint edema in MSU crystal-induced gouty rats was reduced by administration of BERB or Para and that BERB minimized the edema and resulted in faster recovery; as depicted in Fig. 10.

## Conclusions

This study pointed that BERB had a significant inhibitory potential on synovitis and oedema in an experimental gouty arthritis rat model induced by intr-articular injection of acute MSU crystal. This correlated with attenuation of leucocyte influx into the knee and ankle joints, downregulated the expression levels of Elastase, COX-2, MMP-9, and MPO, together with reduced lipid peroxidation, and decreased DNA fragmentation indicating anti-NETosis potential. These therapeutic activities were compared with those attained by treating with the standard drug, Para. In conclusion, BERB mediated significant improvement in MSU-induced inflammation to a similar degree or more effectively than Para. Therefore, BERB is a promising candidate for developing as a novel treatment for GA; however, it is essential to explore the therapeutic activities of BERB in sub-acute and chronic gouty models, such as a gouty model through low-dose and repeated MSU crystal administration. Further well-designed clinical trials are required to confirm its therapeutic efficacy and safety.

## Study limitations

While this study provides significant insights into the anti-arthritic activities of BERB in MSU-induced rats, certain limitations must be acknowledged. First, although the study demonstrates a clear improvement in oxidative stress markers, inflammatory mediators, and functional parameters, it does not comprehensively explore the potential mechanistic pathways in mitigating arthritis; to bridge the gap between pre-clinical investigations and clinical application in the treatment of gouty arthritis. Second, it is recommended to expose all rats of different experimental groups, including negative control rats, to the same stress. However; herein we aimed to compare between the healthy negative control group and the arthritic group, in addition, the treated groups were administrated BERB and Para dissolved in distilled water, and the negative control group already administrated distilled water orally for drinking. Third, more time points after GA induction and during the treatment period should be conducted to better observe progression or recovery over time. Future studies incorporating broader molecular profiling, including pathway-specific modulators, are required to better reveal the full mechanistic axis through which BERB exerts its anti-arthritic potential.

## Supplementary Information

Below is the link to the electronic supplementary material.

Supplementary Material 1

## Data Availability

All data generated or analyzed during this study are included in this published article.

## Abbreviations

ANOVA: Analysis of variance
BERB: Berberine
COX-2: Cyclooxygenase-2
dHL-60 cells: Differentiated HL-60 cells
DPA: Diphenyl amine
GA: Gouty arthritis
GAPDH: Glyceraldehyde-3-phosphate dehydrogenase
H_2_O_2_: Hydrogen peroxide
IL-1β: Interleukin-1 beta
IL-6: Interleukin-6
MCP-1: Monocyte chemotactic protein 1
MDA: Malondialdehyde
MMP-9: Matrix metalloproteinase-9
MPO: Myeloperoxidase
MSU: Monosodium urate
NETs: Neutrophil extracellular traps
NOAEL: No-observed-adverse-effect level
NSAIDs: Non-steroidal anti-inflammatory drugs
OA: Osteoarthritis
Para: Paracetamol
PBS: Phosphate buffered saline
PGE2: Prostaglandin E2
POCD: Post-operative cognitive dysfunction
RA: Rheumatoid arthritis
ROS: Reactive oxygen species
SNI: Spared nerve injury
TCA: Trichloroacetic acid
TNF-α: Tumor necrosis factor-α
VEGF: Vascular endothelial growth factor
