# Supplementary material for: Antiarthritic activities of berberine in a rat model of gouty arthritis
Source: Sci Rep. 2025 Sep 1;15:32153. doi: 10.1038/s41598-025-16622-0 (PMC12402284; doi:10.1038/s41598-025-16622-0)
Supplement: Supplementary file 1 — Supplementary Material 1 [file 41598_2025_16622_MOESM1_ESM.docx]

**Supplementary File:**

**Title:**

**Antiarthritic Activities of Berberine in a Rat Model of Gouty Arthritis**

**
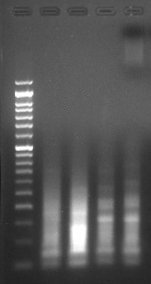
**

**Fig. 9. Effect of treatment on DNA fragmentation detected in knee cells of rats of different experimental groups (full uncropped Gel image)**
